# Whey Protein Combined with Low Dietary Fiber Improves Lipid Profile in Subjects with Abdominal Obesity: A Randomized, Controlled Trial

**DOI:** 10.3390/nu11092091

**Published:** 2019-09-04

**Authors:** Elin Rakvaag, Rasmus Fuglsang-Nielsen, Knud Erik Bach Knudsen, Rikard Landberg, Astrid Johannesson Hjelholt, Esben Søndergaard, Kjeld Hermansen, Søren Gregersen

**Affiliations:** 1 Department of Endocrinology and Internal Medicine, Aarhus University Hospital, 8200 Aarhus, Denmark; 2 Department of Animal Science, Aarhus University, 8830 Tjele, Denmark; 3 Department of Biology and Biological Engineering, Chalmers University of Technology, 412 96 Gothenburg, Sweden; 4 Department of Clinical Medicine, Aarhus University, 8000 Aarhus, Denmark; 5 Steno Diabetes Center Aarhus, 8200 Aarhus, Denmark

**Keywords:** whey protein, dietary fiber, abdominal obesity, postprandial lipemia, triglycerides

## Abstract

Abdominal obesity is associated with elevated postprandial triglycerides (TG), an independent risk factor for cardiovascular diseases. Previous studies show that whey protein (WP) and dietary fiber may separately reduce postprandial TG. However, few studies have investigated the long-term effects of WP and dietary fiber on postprandial TG. We aimed to investigate the separate and combined long-term effects of WP and dietary fiber from wheat bran on postprandial TG and markers of lipid metabolism in subjects with abdominal obesity. We conducted a 12-week, double-blind, randomized, controlled, parallel intervention study. In a 2 × 2 factorial design, 73 adults were randomized to receive 60 g/day of either WP hydrolysate or maltodextrin (MD) combined with high-fiber wheat bran products (HiFi; 30 g dietary fiber/day) or low-fiber refined wheat products (LoFi; 10 g dietary fiber/day). A high-fat meal test was conducted before and after the intervention. Sixty-five subjects were included in the final analyses. There were no differences between intervention groups in postprandial TG assessed as incremental area under the curve (iAUC). WP-LoFi had reduced postprandial TG assessed as total area under the curve (tAUC) and reduced fasting TG compared with all other groups, and reduced fasting apolipoprotein B-48 compared with MD-LoFi. There were no changes in lipoprotein lipase activity. Total cholesterol and apolipoprotein B-100 were reduced after WP intake compared with MD. Total cholesterol was increased after HiFi intake compared with LoFi. In conclusion, intake of WP in combination with low-fiber cereal products for 12 weeks had beneficial effects on postprandial TG tAUC and fasting TG, but not on postprandial TG iAUC in subjects with abdominal obesity. Combining WP with high-fiber wheat bran products did not improve lipid profile.

## 1. Introduction

Abdominal obesity is associated with an atherogenic lipid profile of elevated fasting- and postprandial triglycerides (TG) [1,2,3]. Historically, clinicians have assessed TG in the fasting state; however, postprandial TG is a better predictor of cardiovascular diseases (CVD) than fasting TG [4,5,6]. Abdominal obesity and elevated TG are features of the metabolic syndrome (MetS), a condition characterized by a cluster of risk factors for developing type 2 diabetes (T2D) and CVD [7,8]. The prevalence of abdominal obesity and MetS is increasing worldwide [9,10]. Treatment by lifestyle changes generating weight loss may be effective [11]; however, adherence to complex weight-reducing diets is challenging in the long term [12]. Thus, there is growing interest in the identification of dietary components with the ability to improve lipid metabolism even in the absence of weight reduction. Whey protein (WP), a major constituent of dairy protein, is gaining recognition as a nutritional component with beneficial effects on metabolic risk markers [13,14,15]. Clinical evidence indicates that both immediate and long-term intake of WP supplements may improve lipid profile. Acute studies in overweight or obese subjects [16,17] and subjects with T2D [18] show that supplementing a high-fat meal with WP reduces postprandial TG compared with other protein sources or glucose. Two long-term studies of WP supplementation reported reductions in fasting TG and cholesterol [19,20]. To our knowledge, only one previous study has assessed the long-term effect of WP on postprandial TG [21]. This 12-week intervention found no effects on postprandial TG; however, reduced postprandial levels of apolipoprotein B-48 (apoB-48), a specific marker of TG-rich chylomicron particles, were reported [21]. Another food component of particular interest in relation to metabolic health is dietary fiber. Observational evidence suggests that dietary fiber from cereal products attenuates the risk of MetS, T2D and CVD [22,23,24]. The effects of cereal fiber on lipid metabolism are, however, conflicting. Wheat bran, the fiber-rich outer layer of wheat grain, reduced fasting TG in one short-term study [25], but not in another [26]. Addition of wheat bran to a high-fat meal reduced postprandial TG in an acute study [27]. Six-week supplementation with arabinoxylan, the major dietary fiber component in wheat bran, reduced fasting and postprandial TG in overweight adults compared with placebo [28,29]. This is corroborated by a 12-week study demonstrating a reduction in postprandial TG after consumption of a whole-grain (primarily wheat-based) diet compared with a refined cereal diet [30]. In contrast, a 4-week crossover study with diets rich in arabinoxylan and resistant starch found no effects on fasting or postprandial TG compared with a control diet [31].

In summary, long-term studies of WP or cereal fiber on postprandial TG are sparse, and to our knowledge, no previous studies have investigated the combined effects of WP and cereal fiber on lipid metabolism. 

The aim of our study was to evaluate the effects of a 12-week intervention with WP and high-fiber wheat bran products on postprandial TG (primary outcome) in subjects with abdominal obesity. Secondly, we aimed to investigate potential effects on fasting lipids and markers of lipid metabolism. We hypothesized that the combination of WP supplements and high-fiber cereal products would provide additive effects on reduction of postprandial TG.

## 2. Materials and Methods 

### 2.1. Study Design 

The study was a 12-week, double-blind, randomized, controlled, parallel dietary intervention preceded by 1 week of run-in. Participants were randomly allocated to 1 of 4 groups in a 2 × 2 factorial design: WP + low fiber (WP-LoFi), WP + high fiber (WP-HiFi), maltodextrin (MD) + low fiber (MD-LoFi), or MD + high fiber (MD-HiFi). The allocation was conducted by the investigators using stratified block randomization with blocks of eight, where participants were stratified according to age (2 levels; 40–65 years and 66–99 years) and sex [32]. A department secretary who was not involved in the trial kept the randomization codes in sealed envelopes, ensuring that investigators, laboratory technicians and participants remained blinded throughout the entire study period. 

### 2.2. Study Participants

We recruited 73 participants by advertising through local newspapers and online sites. The study was conducted at the Department of Endocrinology and Internal Medicine at Aarhus University Hospital, Denmark from May 2016 to June 2017. Inclusion criteria were abdominal circumference ≥80 cm (women) or ≥94 cm (men), and age ≥40 years. Exclusion criteria were diabetes, weight change of ≥3 kg within the last 3 months, use of fiber- or protein supplements within the last month, pregnancy/lactation or planned pregnancy, alcohol- or drug abuse, treatment with corticosteroids, any severe renal-, endocrine-, cardiovascular- or psychiatric illness/disease assessed by a physician, blood donation or participation in other clinical trials within the last 3 months. Regular use of medication, including lipid-lowering or antihypertensive medication, was allowed. However, any change in medication one month prior to the study or during the trial was cause for exclusion. All participants gave their informed written consent. The study was conducted in accordance with the Declaration of Helsinki of 1975 as revised in 1983. The study protocol was approved by the Central Denmark Region Committees on Health Research Ethics (Journal no. 1-10-72-370-15). The trial was registered at ClinicalTrials.gov as NCT02931630.

### 2.3. Dietary Intervention

The study participants were instructed to replace habitual intake of bread products (such as bread rolls, rye bread and white bread) and breakfast cereals (such as oatmeal and muesli) with the provided test bread and breakfast cereals. The goal was to consume a daily minimum of six servings of the provided cereal products, equal to 30 g/day of dietary fiber for HiFi, and 10 g/day of dietary fiber for LoFi. A serving of cereal products corresponded to 1 slice of bread or 1.5 dl of breakfast cereals. Participants were free to combine the test bread and breakfast cereals, as they preferred. In those cases where participants did not have a habitual intake of six servings of cereal products per day, they received individual guidance on how to replace other carbohydrate sources in their diet (e.g., rice or pasta). Additionally, participants consumed two daily servings of WP or MD powder. They were instructed to dissolve the powder in approximately 300 mL of cold water and consume the drinks together with a main meal, preferably. The servings for each of the dietary interventions were approximately isoenergetic (Table 1). A nutritionist guided the participants individually on how to incorporate the test products into their habitual diet in order to remain weight stable during the trial. They were instructed to maintain their physical activity level throughout the study. Participants underwent one week of run-in prior to the intervention, during which they were provided with the LoFi test products and MD supplements. Run-in products were coded with separate run-in labels. The run-in allowed for participants to get used to the new food items and learn to incorporate them into their diet. Following run-in, participants were randomized to one of the four diet groups, and the 12-week intervention was initiated. 

### 2.4. Test Products

The nutritional composition of the test products is presented in Table 1. The WP and MD powder supplements were provided by Arla Foods Ingredients Group P/S (Viby, Denmark). The powders were packed in identical, non-transparent sachets. Each serving contained either 30 g of WP hydrolysate (Lacprodan^®^ HYDRO.REBUILD) or 30 g of MD (Glucidex^®^ 19). The wheat bran was provided by Lantmännen Cerealia AB (Malmø, Sweden) and was enzymatically treated with cell wall-degrading enzymes (xylanase, glucanase, cellulase) by DuPont Industrial Biosciences Aps (Brabrand, Denmark), essentially as described by Ingerslev et al. [33]. The enzymatic treatment reduced insoluble arabinoxylan (from 18.5 to 13.7 g/100 g dry matter) and converted it predominantly to arabinoxylan oligosaccharides (AXOS) (from 1.9 to 6.8 g/100 g dry matter) and reduced mixed-linkage β-glucan (from 3.3 to 1.2 g/100 g dry matter). This improved the baking abilities of the wheat bran and allowed for an increased fiber content in the HiFi products [34]. The breads were produced by Lantmännen (Vaasan bakery, Vilnius, Lithuania) and the breakfast cereals were produced by Lantmännen Cerealia AB (Järna, Sweden). The HiFi bread and breakfast cereals were made using equal amounts of enzyme-treated wheat bran and refined wheat. The LoFi products contained refined wheat and were colored with malt extract. The manufacturer took measures to ensure that LoFi and HiFi breads were similar in appearance, texture and packaging. The dietary fiber content of the cereal products and wheat bran was analyzed as described by Knudsen et al. [35], and modified to include the measurements of AXOS [36]. The test breads were kept in freezers at −18 °C. The test products were handed out to the participants at the study visits, and additional products were picked up by the participants when necessary.

### 2.5. Study Visits and Experimental Procedures

The participants received a high-fat, mixed meal test before and at the end of the 12-week intervention. Participants attended the research clinic in the morning after an overnight fast (minimum 10 h). They were instructed to avoid alcohol for 48 h and strenuous physical activity for 24 h prior to the visits. Smoking or caffeine intake was not allowed on the day of the visit. Upon arrival, participants were weighed and a catheter was placed in an antecubital vein for collection of fasting blood samples (t = −15, −10 and 0 min). The basis meal (t = 0 min) consisted of Greek yoghurt (150 g) with muesli (15 g), a croissant (55 g), butter (30 g), full-fat cheese (45 g), sliced cucumber (20 g), and ad libitum tap water. Each basis meal was supplemented with 1.5 slices of HiFi or LoFi bread, as well as one WP or MD powder shake, according to the participant’s intervention group. All meals provided 70 g of fat (55 E%) and a total of approximately 4700 kJ (1125 kcal). WP-LoFi and WP-HiFi meals provided 22 E% protein and 23 E% carbohydrate. MD-LoFi and MD-HiFi meals provided 11 E% protein and 34 E% carbohydrate. The meal was to be consumed within 20 min. We collected postprandial blood samples for TG and free fatty acids (FFA) (t = 60, 90, 120, 240 and 360 min), glucose, insulin and glucagon (t = 15, 30, 60, 90, 120, 240 and 360 min), and apoB-48 and apolipoprotein B-100 (apoB-100) (t = 240 and 360 min). The meal test was repeated after the intervention. Blood samples for measuring total-, LDL and HDL cholesterol as well as alkylresorcinols, a marker of cereal bran intake, were collected in the fasting state. Participants collected 24-h urine samples pre- and post-intervention using 3 L urinary containers kept in cooling bags. Sampling time was noted and volume measured before samples were analyzed for urinary carbamide content (a non-specific marker of protein intake). Before and after the intervention, we performed abdominal subcutaneous adipose tissue biopsies for the measurement of lipoprotein lipase (LPL) activity. The procedures were performed under local analgesia using a Bergström needle. The tissue biopsies were cleaned with saline and snap-frozen in liquid nitrogen before storage at −80 °C. 

Prior to run-in and during week 12, participants were asked to fill in 3-day weighed dietary records (2 weekdays and 1 weekend day) following detailed instructions, and were asked to rate their level of physical activity. A clinical dietician calculated reported energy intakes and nutrient compositions using DANKOST Pro dietary assessment software (Version 1.5.49.21, Copenhagen, Denmark). Participants were required to keep a daily record of their intake of test products in a handed-out journal in order to monitor compliance. Participants met with study personnel at two compliance visits (week 3 and 7), where body weight was monitored and any dietary concerns were discussed. 

### 2.6. Biochemical Analyses

All blood samples for plasma analyses were immediately centrifuged at 2000 × *g* for 15 min at 4 °C. Plasma was then frozen at −20 °C and moved to −80 °C within 8 h. Samples for serum analyses were initially left at room temperature for 30 min, then centrifuged for 10 min at 2000 × *g*. Serum was frozen at −20 °C and moved to −80 °C within 8 hours. Plasma TG, FFA and glucose were determined by standard enzymatic colorimetric assays using commercial kits (TG: cat. 04657594, Roche Diagnostics GmbH), (FFA: cat. 434–91795, Wako chemicals GmbH), (Glucose: cat. 04657527, Roche Diagnostics GmbH). Measurements were performed on a Cobas c111 system. Intra-/inter-assay precision were between 0.8–1.3% and 0.9–3.6% for TG, and between 0.8–1.1% and 0.5–0.6% for glucose. Intra-assay precision for FFA was 1.5%. Plasma insulin was measured by ELISA technique using commercial kits (cat. K6219, Dako Denmark A/S) with an intra-/inter-assay precision of 5.1–7.5% and 4.2–9.3%. Glucagon was measured using radioimmunoassay (cat. GL-32K, EMD Millipore, Darmstadt, Germany). Intra-/inter-assay precisions were 4.0–6.8% and 7.3–13.5%. Serum apoB-48 was measured by a human apoB-48 ELISA kit (no. AKHB48, Shibayagi Co., Ltd., Shibukawa, Gunma, Japan) with an intra-/inter-assay precision of 1.5–2.2% and 7.4–15.9%. Serum apoB-100 was measured by a human apoB ELISA pro kit (cat. 3715-1HP-2, Mabtech AB, Nacka Strand, Sweden) with an intra-/inter-assay precision of 2.0% and 10.0%. Plasma alkylresorcinol homologues C17:0, C19:0, C21:0, C23:0, C25:0 and their sum (total alkylresorcinols) were analyzed by gas chromatography-mass spectrometry (GC-MS), as described by Wierzbicka et al. [37]. Samples and reference standards were analyzed on GC–MS (Finnigan TM Trace GC Ultra Gas chromatograph coupled to a Finnigan Trace DSQ II mass detector, Thermo Fisher Scientific, Waltham, MA, USA). The within-batch coefficient of variation was <10% and the between-batch coefficient of variation was <15% for total alkylresorcinols. Total cholesterol, HDL and LDL were analyzed at the Department of Clinical Biochemistry at Aarhus University Hospital (DS/EN ISO 15189:2013 approved). Urinary carbamide was analyzed at the Department of Clinical Biochemistry at Aarhus University Hospital (DS/EN ISO 15189:2013 approved). Heparin-releasable LPL activity was measured in 30–50 mg adipose tissue biopsy samples by the glycerol-stabilized method described by Nilsson-Ehle and Schotz [38]. Data are expressed as μmol FFA per hour per gram lipid. In short, the tissue was defrosted and incubated in 5 U/mL heparin elution buffer (cat. no. H-0777; Sigma, St. Louis, MO, USA) for 45 min at room temperature. Afterward, the eluant was incubated in ^3^H-triolein-containing substrate (0.5 mCi/mL, NET-431; Perkin Elmer, Waltham, Massachusetts, USA) for 2 h at 37 °C (duplicate determinations). The reaction was stopped using methanol: chloroform: heptane (34:38:28), and after centrifugation, the supernatant was counted on a scintillation counter. The inter-assay coefficient of variation was 11.0%.

### 2.7. Calculations and Statistical Analyses

In order to estimate the sample size, we performed a pre-study sample size calculation based on a previous study from our group [17], using TG iAUC as reference. In order to detect a between-group difference in TG iAUC of 110 mmol/L × 360 min (80% power, α = 0.05), 66 participants were needed, assuming an average SD of 155 mmol/L × 360 min. Accounting for an expected dropout rate of 20%, we aimed to enroll 80 participants in total. All statistical analyses were performed using STATA/IC 15.1 (StataCorp LP College Station, TX, USA). Only participants who completed the trial successfully were included in the final analyses. AUCs were calculated by the trapezoidal method. A two-factor ANOVA model was used to assess treatment effects of protein level (high vs. low) and fiber level (high vs. low), as well as interactions between the protein- and fiber levels. If a main effect or interaction was detected, we conducted a pairwise comparison of groups corrected for multiple comparisons using the Tukey–Kramer method. Normal distribution and homogeneity of variance were checked by diagnostic plots of residuals (quantile-quantile plots, histograms and residuals-vs-fitted plots). All estimates were adjusted for sex and age and reported as means and 95% CI, unless otherwise stated. In cases where the assumptions of normality and equal variance across groups were invalid, the dependent variable was log-transformed. Postprandial TG responses within each group were analyzed by repeated-measures ANOVA (reported in supplementary Figure S1). One-way ANOVA with Bonferroni corrections for multiple comparisons was applied to assess differences in energy- and macronutrient intakes from the test products. Changes in physical activity level were assessed by Wilcoxon signed rank test (within-group changes) and Chi-square test (between groups). *P* < 0.05 was considered statistically significant.

## 3. Results

### 3.1. Baseline Characteristics

**Figure 1:**
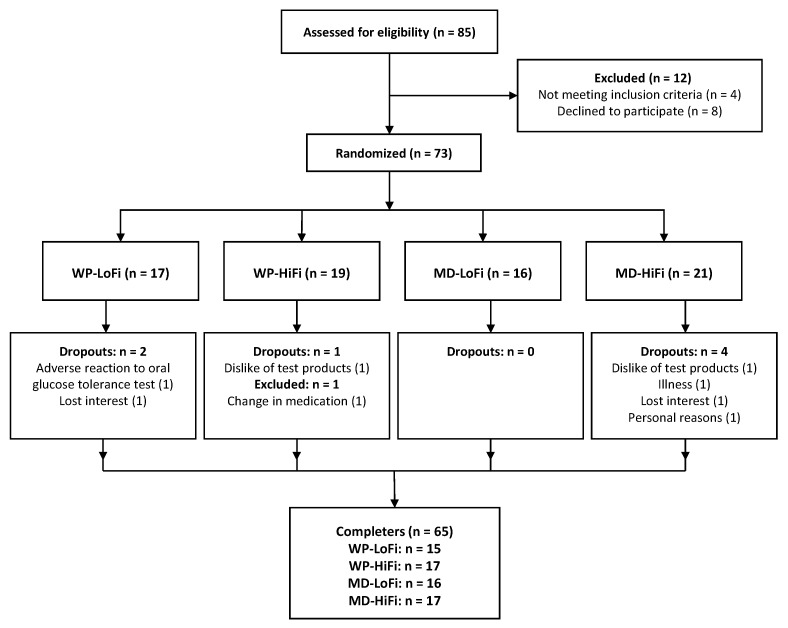
Participant flow diagram. HiFi, high fiber; LoFi, low fiber; MD, maltodextrin, WP, whey protein.

The trial was terminated when 66 participants had completed all experimental visits. However, one participant was later excluded from all analyses due to longer-term treatment with antibiotics. Dropout rate was 10% (Figure 1). Baseline characteristics of the 65 subjects who completed the study are presented in Table 2.

### 3.2. Compliance

The average intervention duration was 84 days in all diet groups. The self-reported consumption of test products and corresponding nutrient intakes are presented in Table 3. Participants consumed on average 5.3 (95% CI: 5.1–5.5) servings/day of the cereal products, corresponding to 88% of the target, and on average 94% (95% CI: 92–96%) of the test powders (no differences between groups). 

Self-reported physical activity level remained unchanged throughout the intervention period for all groups (data not shown). Body weight, plasma alkylresorcinols and urinary carbamide measurements are presented in Table 4. There was no change in body weight for any of the groups. Plasma alkylresorcinols increased in both of the HiFi groups, and decreased for WP-LoFi. Urinary carbamide excretion increased for both WP groups, increased modestly for MD-HiFi, and was unchanged for MD-LoFi. 

### 3.3. Dietary Intake

Self-reported dietary intake at baseline and end of intervention is shown in Table 5. There was no difference in energy intake between groups. As expected, both WP groups reported an increased protein intake, leading to a mean difference between WP and MD of 55 g/day (95% CI: 47, 62 g/day, *p* < 0.001). MD-HiFi also reported a higher protein intake at 12 weeks, which is supported by the concomitant increase in urinary carbamide. Total dietary fiber intake increased in both of the HiFi groups and decreased in the LoFi groups, leading to a mean difference of 19 g/day (95% CI: 16, 21 g/day, *p* < 0.001). There were no differences between groups in terms of fat intake.

### 3.4. Lipid Profile

Fasting and postprandial values of TG, apoB-48, apoB-100, FFA, fasting cholesterols and adipose tissue LPL activity before and after the 12-week intervention are presented in Table 6. There was an interaction between protein and fiber level for TG iAUC (primary outcome), but there were no differences between groups after correction for multiple comparisons. We found a main effect of protein and an interaction between protein and fiber for postprandial TG total area under the curve (tAUC). TG tAUC was reduced for WP-LoFi and WP-HiFi compared with baseline, although only the reduction for WP-LoFi differed from the other groups. No differences were found for TG tAUC between WP-HiFi and the two MD groups. For fasting TG, we also observed a main effect of protein and an interaction between protein and fiber; WP-LoFi had a greater reduction in fasting TG at 12 weeks compared with all other groups, and compared with baseline. Postprandial TG responses are presented for each group and meal test in supplementary Figure S1. 

We found an interaction between protein and fiber on fasting and postprandial apoB-48. Fasting apoB-48 was reduced for WP-LoFi compared with MD-LoFi, while no differences between groups were found for apoB-48 tAUC. There was a main effect of protein on fasting apoB-100; WP groups had a greater reduction in fasting apoB-100 than MD groups (−10.4 mg/dL; 95% CI: −17.3, −3.5 mg/dL, *p* = 0.004). Finally, we found main effects of protein and fiber on total cholesterol; WP groups had a greater reduction in total cholesterol (−0.38 mmol/L; 95% CI: −0.60, −0.17 mmol/L, *p =* 0.001) than MD groups. There was an increase in total cholesterol for HiFi compared with LoFi (0.23 mmol/L; 95% CI: 0.01, 0.44 mmol/L, *p =* 0.04). There were no differences between interventions in fasting and postprandial FFA, apoB-100 tAUC, HDL and LDL cholesterol, or adipose tissue LPL activity. Additional adjustment for statin treatment did not change any of the results, except for LDL cholesterol, where adjusting for statins resulted in a main effect of fiber; LDL cholesterol was increased for HiFi compared with LoFi (0.20 mmol/L; 95% CI: 0.02, 0.38 mmol/L, *p =* 0.03). 

### 3.5. Glucose, Insulin and Glucagon

Fasting and postprandial values for glucose, insulin and glucagon are listed in Table 7. There was a main effect of fiber level on glucose iAUC and glucagon iAUC. HiFi groups had an increase in glucose iAUC of 96 mmol/L × 360 min (95% CI: 5, 188 mmol/L × 360 min, *p* = 0.04) and a reduced glucagon iAUC of −2228 pg/mL × 360 min (95% CI: −4353, −104 pg/mL × 360 min, *p* = 0.04). There were no effects on fasting glucose and glucagon, or fasting and postprandial insulin.

## 4. Discussion

In the present study, we found no effects on TG iAUC; however, we found reductions in fasting TG and postprandial TG assessed as tAUC after 12-week intake of WP supplements. The reductions were most pronounced in the group receiving WP in combination with low-fiber cereal products. To our knowledge, an effect of long-term WP intake on postprandial TG has not previously been reported. Not many studies exist in this area; a previous study from our group found no changes in postprandial TG following 12-week intake of WP isolate, despite using a similar dose of protein [21]. In the previous study, the WP intervention was combined with a milk fat intervention, and the participants gained weight; this may have diminished a potential effect on postprandial TG [21]. 

Currently, there is no commonly agreed definition of elevated postprandial TG, or of how this should be assessed in a standardized manner. In an attempt to unify postprandial lipemia assessments, an expert panel statement defined a desirable postprandial TG as below 2.5 mmol/L at any time point following an oral fat challenge [6]. In the present study, 12-week intake of WP-LoFi reduced the average TG concentrations from above to below this limit at 4 h and 6 h of the high-fat meal test (Figure S1). 

WP-LoFi had reduced TG tAUC, while there were no effects on TG iAUC. In clinical research, the calculation of AUC is a common approach to simplify data by combining multiple readings into a single value for each subject [39]. While iAUC correlates more strongly with the rise in TG after a fat load, tAUC correlates strongly with fasting TG [40], and has a higher reproducibility in fat tolerance tests than iAUC [41]. In the present study, the reduction in TG tAUC for WP-LoFi appeared to be particularly driven by a reduction in fasting TG. A reduction in fasting TG following WP consumption was demonstrated in a recent eight-week study [19], as well as in a meta-analysis of previous studies [42]. It is important to note that most of our participants had fasting TG below the MetS criteria of 1.7 mmol/L [8]. Although not significantly different between groups, the baseline fasting TG levels tended to be higher in the WP-LoFi group (1.63 mmol/L) than in the other groups (0.93–1.31 mmol/L), thus allowing for a greater possibility to observe a reduction. The clinical implications of lowering fasting TG in normolipemic individuals are unknown. Nonetheless, we observed an almost 40% reduction in fasting TG following the WP-LoFi intervention—an effect which may be of clinical significance if extrapolated to high-risk individuals with hypertriglyceridemia [43]. 

Reductions in plasma TG levels may be due to decreased chylomicron secretion from the enterocytes, increased TG clearance from the blood, decreased endogenous VLDL production, or a combination. The number of circulating chylomicron particles is reflected in apoB-48, which is synthesized by the intestine and represents the diet-derived lipids. In the fasting state, apoB-48 is a marker of atherogenic chylomicron remnants [44], and is correlated with the prevalence of CVD [45]. We found a reduction in fasting apoB-48; however, we found no change in postprandial apoB-48, in contrast to what was previously reported after 12-week intake of WP compared with casein [21]. ApoB-100 reflects the number of circulating hepatic-derived lipoproteins [46]. In the present study, fasting apoB-100 and total cholesterol were reduced after WP intake, while LDL was unchanged. This finding is in agreement with previous studies [19,20], and indicates a decreased hepatic synthesis of VLDL and VLDL remnants. Clearance of TG from chylomicrons and VLDL is mainly dependent on LPL activity within adipose tissue. LPL is primarily stimulated by insulin [47]. Evidence from acute studies indicates that WP may exert beneficial metabolic effects via an insulinotropic property [48]. However, we did not find any differences in insulin levels or LPL activity between groups. Thus, the observed reduction in TG for WP-LoFi likely reflects a reduced endogenous VLDL production along with a reduced chylomicron secretion from the intestine.

Since plasma concentrations of TG and apoB-48 may be reduced by statin treatment [49], it may be argued that use of lipid-lowering medication should have been an exclusion criteria in our study. However, it is important to note that our participants did not change medication doses throughout the study, and that adjusting for statin treatment did not affect the results.

We found an increase in the postprandial glucose response after 12 weeks of HiFi consumption compared with LoFi, along with a decreased postprandial glucagon response. This combination might suggest impaired insulin sensitivity, in contrast to what has previously been reported for high cereal fiber interventions [50]. However, there was no compensatory increase in insulin levels, which is normally the primary reaction upon developing insulin resistance. 

Unexpectedly, the reduction in TG was more pronounced when WP was combined with LoFi rather than with HiFi products. Interestingly, a previous study has suggested that cereal fibers may interfere with protein digestion and/or absorption in the small intestine [50]. Biomarkers of protein intake (urinary nitrogen: creatinine ratio and fecal isovaleric acid) indicated a reduced protein absorption when a high-protein diet was combined with high cereal fiber [50]. This effect might explain the differences observed between the WP groups in our study; however, the 24-h urinary carbamide measurements in both groups concurred with the reported protein intake, which does not suggest an interference by cereal fiber. Another study recently reported a surprising increase in postprandial TG following 12-week consumption of wholegrain wheat compared with refined wheat [51]. If long-term wholegrain wheat consumption may in fact increase postprandial TG, this might also explain the difference in TG between WP-LoFi and WP-HiFi. However, we did not see an increase in TG response for MD-HiFi, which contradicts such an effect of wheat bran intake. The discordant results may be due to differences in the dietary fiber intake; in the present study, the HiFi participants consumed a larger amount of dietary fiber from the test products than the participants in the previous study [51]. 

In contrast to what was previously reported after 12-week intake of a comparable amount of cereal fiber [30], we did not observe any improvement in postprandial TG after the HiFi intervention. It should be noted that the previous study was conducted in individuals with the MetS [30], while in the present study, only half of the participants fulfilled the criteria for MetS. The discordant results may also relate to differences in fiber sources. The previous study used wholegrain wheat, but also wholegrain products from barley, oat and rye [30]. The wheat bran used for the HiFi products in our study was enzymatically treated, modifying some of the physicochemical properties of the bran constituents. The major modification was the conversion of insoluble arabinoxylan (the main fiber constituent of wheat) to the more soluble AXOS. While there is a general consensus that soluble cereal fiber may lower cholesterol [52], the evidence of a cholesterol-lowering effect of wheat—which consists mostly of insoluble fiber—is inconsistent [53,54]. The effect of enzyme-treated wheat bran on cholesterol levels is unknown. Surprisingly, we observed an increase in total cholesterol and LDL cholesterol (when adjusting for statin treatment) for HiFi compared with LoFi. It should be noted that the HiFi products contained more fat and protein than the LoFi products, which may have affected the results.

Strengths of the current study are the double-blind, randomized design, a low dropout rate, and the fact that we succeeded in maintaining weight stability in all intervention groups. In addition, we included biochemical compliance markers of cereal fiber- and protein intake. These markers were well aligned with the self-reported intake data, and indicated excellent compliance with our study diets. The study also has some limitations; using WP supplements to create the high-protein intervention implies a compensation in other macronutrients in order to maintain an isoenergetic diet. This makes it difficult to discern whether effects are caused by the WP supplementation, or by changes in the macronutrient composition of the diet, or a combination of both. Based on the dietary records, WP-HiFi reported a displacement of carbohydrate with protein, where carbohydrate was reduced from 46 E% to 35 E%. High protein-low carbohydrate diets may be a strategy for controlling TG levels in healthy individuals [55]. While changes in carbohydrate intake differed between the groups in our study (not surprisingly, when using MD as control), WP-LoFi did not report a significant change in E% from carbohydrates, indicating that the reductions in plasma lipids observed within this group are not a result of reduced carbohydrate intake. However, there was an indication of a displacement of fat with protein for WP-LoFi, where fat intake went from 37 E% to 29 E% (although not statistically significant). This should be taken into account when assessing the changes in plasma lipids for WP-LoFi. In this regard, it is important to note that the change in fat intake did not significantly differ between the groups in our study, and may therefore not explain the differential effects on plasma lipids.

The use of MD as the only comparison to WP is a limitation of our study. It would be interesting to compare the WP intervention with another protein source as well, in order to determine whether the effects are specific to WP or high-protein diets in general. Interestingly, a previous 12-week study found reductions in TG and cholesterol levels after WP, but not casein supplementation, despite similar macronutrient compositions in the two groups [20]. This indicates that the effects were not solely attributed to the modification of macronutrients. In addition, it would be interesting to test our intervention products in an ad libitum setting.

Another limitation is that we did not provide the participants with a standardized meal on the evening prior to the meal tests. However, we diminished the impact of several potential confounding factors prior to the test days by using a 10-h fast, and by instructing participants to avoid strenuous physical activity and alcohol intake. Based on the power calculation, we intended to include 66 participants. Unfortunately, we only managed to include 65 participants in the final analyses. We cannot exclude that this may have affected our results. Furthermore, we cannot exclude the possibility that unobserved metabolic processes or parameters may have influenced our results and the relative small sample size in our study must be considered critically. It should also be noted that the reported fiber intake at baseline (22 g/d) was comparatively higher than in many populations [56]; however, it was similar to the average intake of dietary fiber in the Danish population [57]. 

## 5. Conclusions

In conclusion, intake of WP supplements for 12 weeks had beneficial effects on the fasting and postprandial lipid profile in subjects with abdominal obesity, but there were no effects on TG iAUC. Contrary to our hypothesis, the beneficial effects were greater when WP supplements were combined with low-fiber products than with high-fiber wheat bran products. 

Future studies are needed in order to clarify whether cereal fiber interferes with protein absorption and/or digestion. In addition, future studies should aim to clarify whether WP exerts beneficial effects on lipid metabolism in the absence of changes in the macronutrient composition. We also recommend that future studies address whether patients with hypertriglyceridemia may benefit from daily WP intake.

## Figures and Tables

**Table 1:** Nutritional composition of test products (content per serving).

	Bread		Breakfast Cereals		Whey Protein	Malto-dextrin
	High Fiber	Low Fiber	High Fiber	Low Fiber		
Serving size (g)	40	33	33	29	36	34
Energy (kJ)	371	359	455	443	527	527
Available carbohydrate (g)	11.0	15.5	18.6	21.6	0.4	31.0
Starch (g)	9.3	15.2	18.2	21.5	-	-
Sugars (g)	1.7	0.4	0.5	0.2	-	-
Protein (g)	6.1	3.3	3.9	3.2	30.1	0.2
Fat (g)	1.2	0.8	0.8	0.3	0.4	0.1
Total dietary fiber (g)	4.4	1.4	5.4	1.2	0	0

**Table 2:** Baseline characteristics of study participants.

	WP-LoFi (*n* = 15)	WP-HiFi (*n* = 17)	MD-LoFi (*n* = 16)	MD-HiFi (*n* = 17)
Age, years ^1^	67 (60–69)	65 (59–68)	62 (58–68)	64 (56–67)
Sex, *n* (male/female)	9/6	7/10	8/8	7/10
BMI, kg/m^2^	28.4 (4.1)	29.6 (2.3)	30.3 (4.5)	29.1 (3.6)
Waist circumference, cm (male/female)	105 (10)/91 (7)	108 (8)/93 (6)	105 (7)/100 (12)	109 (8)/90 (7)
Drug treatment for dyslipidemia, *n*	4	1	6	3
Drug treatment for hypertension, *n*	5	6	3	3
Metabolic syndrome, *n* (%)	10 (67)	9 (53)	8 (50)	7 (41)

All values are presented as means (SD), unless otherwise specified. ^1^ Median (25th and 75th centile). HiFi, high fiber; LoFi, low fiber; MD, maltodextrin; WP, whey protein.

**Table 3:** Self-reported intake of test products (amount and nutrient intake) during the 12-week intervention.

	WP-LoFi (*n* = 15)	WP-HiFi (*n* = 17)	MD-LoFi (*n* = 14)	MD-HiFi (*n* = 17)	*p* Value
Bread (g/day)	146.9 (28.0)	174.6 (44.4)	165.6 (33.1)	166.5 (32.1)	0.17
Breakfast cereals (g/day)	24.4 (21.2)	34.5 (28.1)	13.1 (16.2)	33.7 (24.5)	0.05
Powder (sachets/day)	1.83 (0.14)	1.87 (0.24)	1.96 (0.12)	1.86 (0.16)	0.25
Energy (kJ/day)	2941 (405)	3078 (333)	3017 (265)	2971 (407)	0.72
Protein (g/day)	72.4 (5.9) ^a^	86.7 (9.9) ^b^	18.1 (2.4) ^c^	29.3 (4.8) ^d^	<0.01
Fat (g/day)	4.4 (0.6) ^a^	7.0 (1.0) ^b^	4.2 (0.6) ^a^	6.2 (1.0) ^c^	<0.01
Carbohydrate (g/day)	88.4 (17.5) ^a^	68.4 (10.0) ^b^	147.7 (12.1) ^c^	121.9 (15.8) ^d^	<0.01
Dietary fiber (g/day)	7.2 (1.3) ^a^	25.0 (3.2) ^b^	7.5 (1.0) ^a^	24.0 (4.4) ^b^	<0.01

Values are means (SD). Intake of study products based on self-reported daily journals. Two participants failed to complete the journals; *N* = 63. Nutrient intake calculations based on chemical analysis of test products. Different superscript letters within a row indicate statistically significant differences between groups (*p* < 0.05); analyzed using one-way ANOVA with Bonferroni adjustments for multiple comparisons. HiFi, high fiber; LoFi, low fiber; MD, maltodextrin; WP, whey protein.

**Table 4:** Body weight and biochemical markers of compliance at baseline (week 0) and end of intervention (week 12).

	WP-LoFi (*n* = 15)		WP-HiFi (*n* = 17)		MD-LoFi (*n* = 16)		MD-HiFi (*n* = 17)		*p* Value, ∆
	Baseline	Week 12	Baseline	Week 12	Baseline	Week 12	Baseline	Week 12	
Body weight (kg) ^1^	83.9 (14.6)	83.8 (15.3)	87.9 (13.2)	88.5 (12.9)	89.9 (14.0)	90.5 (14.0)	81.7 (15.1)	82.3 (15.3)	0.38
Alkylresorcinols (nmol/L) ^2^	46.3 (30.4–56.6)	27.2 (22.7–37.4) *^,a^	32.8 (27.3–39.9)	179.9 (84.2–246.5) *^,b^	37.6 (24.7–62.7)	40.8 (25.6–75.1) ^a^	46.7 (30.1–58.4)	226.8 (150.4–280.3) *^,b^	<0.01
Urinary carbamide excretion (mmol/24 h) ^1^	369 (103)	576 (156) *^,a^	311 (114)	534 (144) *^,a^	396 (150)	368 (91) ^b^	315 (132)	373 (175) *^,b^	<0.01

^1^ Mean (SD). ^2^ Median and centiles (25th–75th). Overall *p* Value indicates a difference in ∆ (week 12–baseline), assessed by one-way ANOVA. Different superscript letters within a row indicate significant difference in Δ (week 12–baseline) between groups after Tukey correction (*p* < 0.05). * Significantly different from baseline (*p* < 0.05). HiFi, high fiber; LoFi, low fiber; MD, maltodextrin; WP, whey protein.

**Table 5:** Total energy- and macronutrient intakes at baseline (week 0) and end of intervention (week 12) based on self-reported 3-day dietary records.

	WP-LoFi (*n* = 15)		WP-HiFi (*n* = 17)		MD-LoFi (*n* = 16)		MD-HiFi (*n* = 16)		*p* Value, ∆
	**Baseline**	Week 12	Baseline	Week 12	Baseline	Week 12	Baseline	Week 12	
Energy (kJ/day)	8325 (2261)	8230 (1603)	9242 (2837)	8827 (1787)	8812 (1850)	8890 (2141)	8642 (2593)	10016 (2226) *	0.17
Protein (g/day)	79.1 (20.0)	133.3 (17.1) *^,a^	83.1 (15.9)	149.7 (11.2) *^,a^	85.9 (22.8)	78.7 (14.8) ^b^	79.4 (18.0)	94.9 (17.8) *^c^	<0.01
Protein (E%)	17 (3)	28 (4)	16 (3)	30 (5)	17 (3)	15 (2)	16 (3)	16 (2)	
Carbohydrate (g/day)	203.3 (84.9)	184.6 (46.6) ^a, b^	254.2 (91.3)	183.0 (54.5) *^,a^	224.1 (66.7)	254.0 (52.8) ^b, c^	217.8 (64.1)	274.7 (62.1) *^,c^	<0.01
Carbohydrate (E%)	40 (8)	38 (7)	46 (9)	35 (6)	43 (10)	49 (7)	43 (7)	47 (6)	
Fat (g/day)	83.5 (25.4)	66.8 (26.5)	83.4 (34.9)	73.6 (30.3)	84.2 (27.3)	76.0 (34.2)	85.6 (36.6)	86.5 (36.8)	0.50
Fat (E%)	37 (7)	29 (7)	33 (8)	30 (8)	35 (8)	31 (9)	36 (7)	31 (7)	
SFA (g/day)	31.8 (13.6)	28.0 (14.7)	28.3 (14.3)	27.8 (15.2)	29.8 (12.3)	27.6 (16.3)	29.3 (14.1)	31.0 (16.6)	0.74
Dietary fiber (g/day)	20.2 (8.2)	15.0 (4.6) *^,a^	22.7 (4.9)	33.7 (4.8)*^,b^	23.4 (6.4)	16.9 (5.6) *^,a^	24.4 (8.8)	35.6 (4.8) *^,b^	<0.01

Values are means (SD). One participant failed to complete the dietary records; *N* = 64. Overall *p* Value indicates a difference in ∆ (week 12–baseline), assessed by one-way ANOVA. Different superscript letters within a row indicate significant difference in Δ (week 12–baseline) between groups after Tukey correction (*p* < 0.05). * Significantly different from baseline (*p* < 0.05). HiFi, high fiber; LoFi, low fiber; MD, maltodextrin; WP, whey protein; E%, energy percentage.

**Table 6:** Postprandial and fasting TG, apoB-48, apo-B100, FFA, fasting cholesterols and LPL activity at baseline and week 12.

	WP-LoFi (*n* = 15)		WP-HiFi (*n* = 17)		MD-LoFi (*n* = 16)		MD-HiFi (*n* = 17)		Two-Factor ANOVA, *p* ^6^		
	Baseline	Week 12	Baseline	Week 12	Baseline	Week 12	Baseline	Week 12	Protein Level	Fiber Level	Inter-Action
TG iAUC (mmol/L × 360 min) ^1^	276 (132)	223 (99)	232 (95)	208 (65)	234 (108)	292 (159)	310 (217)	274 (150)	0.11	0.24	0.04
TG tAUC (mmol/L × 360 min) ^2^	802 (560–1048)	544 (424–681) *^,a^	615 (565–907)	601 (517–753) *^,b^	790 (488–1053)	816 (550–1105) ^b^	622 (491–1051)	583 (508–892) ^b^	**<0.01**	0.64	<0.01
ApoB–48 tAUC (mg/L × 360 min) ^2^	2800 (2066–3683)	2144 (1424–2672) *	4162 (2596–5459) ^3^	3555 (1915–5294) ^3^	3002 (1988–4408)	2563 (1837–5261)	3667 (2518–4324)	2923 (2626–4118)	0.14	0.59	0.04
ApoB-100 tAUC (mg/dL × 360 min) ^1^	27317 (7496)	25315 (9081) *	28638 (5416)	27305 (5426)	26664 (10864)	26393 (10987)	24015 (10762)	24784 (8336)	0.06	0.26	0.95
FFA tAUC (mmol/L × 360 min) ^1^	117 (22)	117 (33)	126 (31)	125 (25)	120 (34)	119 (29)	114 (35)	125 (30)	0.49	0.49	0.45
Fasting TG (mmol/L) ^2^	1.63 (1.03–1.90)	0.85 (0.76–1.11) *^,a^	1.16 (1.06–1.51)	1.05 (0.84–1.24) ^b^	1.31 (0.81–1.87)	1.22 (0.98–2.47) ^b^	0.93 (0.79–1.25)	0.90 (0.76–1.37) ^b^	**<0.01**	0.17	**<0.01**
Fasting apoB-48 (mg/L) ^2^	4.2 (3.0–5.3)	2.8 (1.7–3.7) *^,a^	6.9 (3.8–10.8)	6.4 (3.0–10.4) ^a, b^	6.4 (3.5–7.8)	4.5 (3.1–11.3) ^b^	6.7 (5.5–7.8)	5.5 (3.8–7.3) ^a, b^	0.06	0.59	**<0.01**
Fasting apoB-100 (mg/dL) ^1^	77.5 (19.7)	69.9 (23.8) *^,^^a^	83.8 (15.6)	78.5 (15.9) ^a, b^	74.4 (30.4)	75.2 (32.0) ^a, b^	64.1 (35.0)	70.9 (23.2) *^,b^	**<0.01**	0.16	0.66
Fasting FFA (mmol/L) ^1^	0.39 (0.12)	0.39 (0.15)	0.44 (0.15)	0.41 (0.12)	0.42 (0.09)	0.37 (0.12)	0.36 (0.16)	0.39 (0.12)	0.89	0.39	0.17
Total cholesterol (mmol/L) ^1^	5.2 (0.8)	5.0 (0.8) *^,a^	5.5 (1.0)	5.4 (1.0) ^a^	5.3 (1.3)	5.3 (1.3) ^a, b^	5.1 (1.1)	5.4 (1.0) *^,b^	**<0.01**	**0.04**	0.78
LDL (mmol/L) ^2^	3.4 (2.5–3.6)	3.3 (2.2–3.8)	3.7 (3.3–4.0)	3.4 (3.2–3.9)	3.6 (2.3–3.9)	3.3 (1.9–3.7) ^4^	2.9 (2.3–3.6)	3.2 (2.7–3.9) *	0.09	0.08	0.07
HDL (mmol/L) ^2^	1.3 (1.1–1.6)	1.4 (1.2–1.9) *	1.3 (1.2–1.6)	1.3 (1.2–1.5)	1.2 (1.1–1.5)	1.3 (1.0–1.6)	1.5 (1.3–1.7)	1.6 (1.3–2.0) *	0.85	0.84	0.08
LPL activity (μmol FFA/h/g) ^2^	1.9 (1.5–2.2) ^5^	1.6 (1.3–2.7) ^5^	2.3 (1.2–2.6) ^5^	2.1 (1.0–4.0) ^5^	1.6 (1.1–2.0) ^5^	1.7 (0.9–2.4) ^5^	1.3 (0.9–1.8) ^3^	1.5 (0.8–2.0) ^3^	0.80	0.79	0.55

^1^ Mean (SD). ^2^ Median (25th–75th centile). ^3^ *n* = 16. ^4^ *n* = 15. ^5^ *n* = 13. ^6^ *P* values indicate overall main effects and interactions between protein and fiber levels. Different superscript letters within a row indicate significant difference in Δ (week 12–baseline) between groups after Tukey correction (*p* < 0.05). * Significantly different from baseline (*p* < 0.05). Data were log-transformed for analyses of TG tAUC and apoB-48 tAUC. TG, triglycerides; apoB-48, apolipoprotein B-48; apoB-100, apolipoprotein B-100; FFA, free fatty acids; LPL, lipoprotein lipase; HiFi, high fiber; iAUC, incremental area under the curve; LoFi, low fiber; MD, maltodextrin; WP, whey protein.

**Table 7:** Postprandial and fasting glucose, insulin and glucagon at baseline and week 12.

	WP-LoFi (*n* = 15)		WP-HiFi (*n* = 17)		MD-LoFi (*n* = 16)		MD-HiFi (*n* = 17)		Two-factor ANOVA, *p*^3^		
	Baseline	Week 12	Baseline	Week 12	Baseline	Week 12	Baseline	Week 12	Protein Level	Fiber Level	Inter-Action
Glucose iAUC (mmol/L × 360 min) ^1^	54 (180)	49 (78)	45 (107)	105 (138)	171 (239)	65 (180) *	84 (231)	105 (153)	0.13	0.04	0.50
Insulin iAUC (pmol/L × 360 min) ^2^	67,996 (53,001–90,413)	68,804 (45,510–88,895)	64,370 (52,105–77,430)	61,526 (48,408–79,128)	66,701 (383,75–117,323)	62,495 (44,173–114,925)	61,093 (42,746–82,741)	48,267 (39,350–81,683)	0.34	0.21	0.74
Glucagon iAUC (pg/mL × 360 min) ^1^	11,671 (4430)	12,691 (2427)	13,958 (6855)	11,477 (5983) *	3567 (3625)	4389 (3069)	5901 (3046)	5713 (2683)	0.31	0.04	0.24
Fasting glucose (mmol/L) ^1^	5.6 (0.3)	5.6 (0.4)	5.5 (0.6)	5.7 (0.6) *	5.6 (0.3)	5.8 (0.4)	5.5 (0.4)	5.6 (0.6)	0.52	0.39	0.20
Fasting insulin (pmol/L) ^2^	50 (36–75)	47 (35–69)	44 (31–48)	43 (30–49)	45 (32–69)	51 (41–82)	31 (24–56)	31 (23–46)	0.48	0.68	0.26
Fasting glucagon (pmol/mL) ^1^	64 (16)	64 (16)	63 (17)	66 (20)	63 (16)	64 (17)	61 (17)	65 (16)	0.90	0.31	0.89

^1^ Values are means (SD). ^2^ Values are medians and centiles (25th–75th).^3^ *p* Values indicate overall main effects and interactions between protein and fiber levels. * Significantly different from baseline (*p* < 0.05). Data were log-transformed for insulin iAUC. WP, whey protein; LoFi, low fiber; HiFi, high fiber; MD, maltodextrin.

## Supplementary Materials

The following are available online at https://www.mdpi.com/2072-6643/11/9/2091/s1, Figure S1: Postprandial triglyceride responses (mmol/L) after intake of a high-fat meal at week 0 and week 12 by intervention group.
