# Supplementary material for: Whey Protein Combined with Low Dietary Fiber Improves Lipid Profile in Subjects with Abdominal Obesity: A Randomized, Controlled Trial
Source: Nutrients. 2019 Sep 4;11(9):2091. doi: 10.3390/nu11092091 (PMC6770182; doi:10.3390/nu11092091)
Supplement: Supplementary file 1 [file nutrients-11-02091-s001.pdf]

Figure S1. Postprandial triglyceride responses (mmol/L) after intake of a high-fat meal at week 0 and week 12 by intervention group

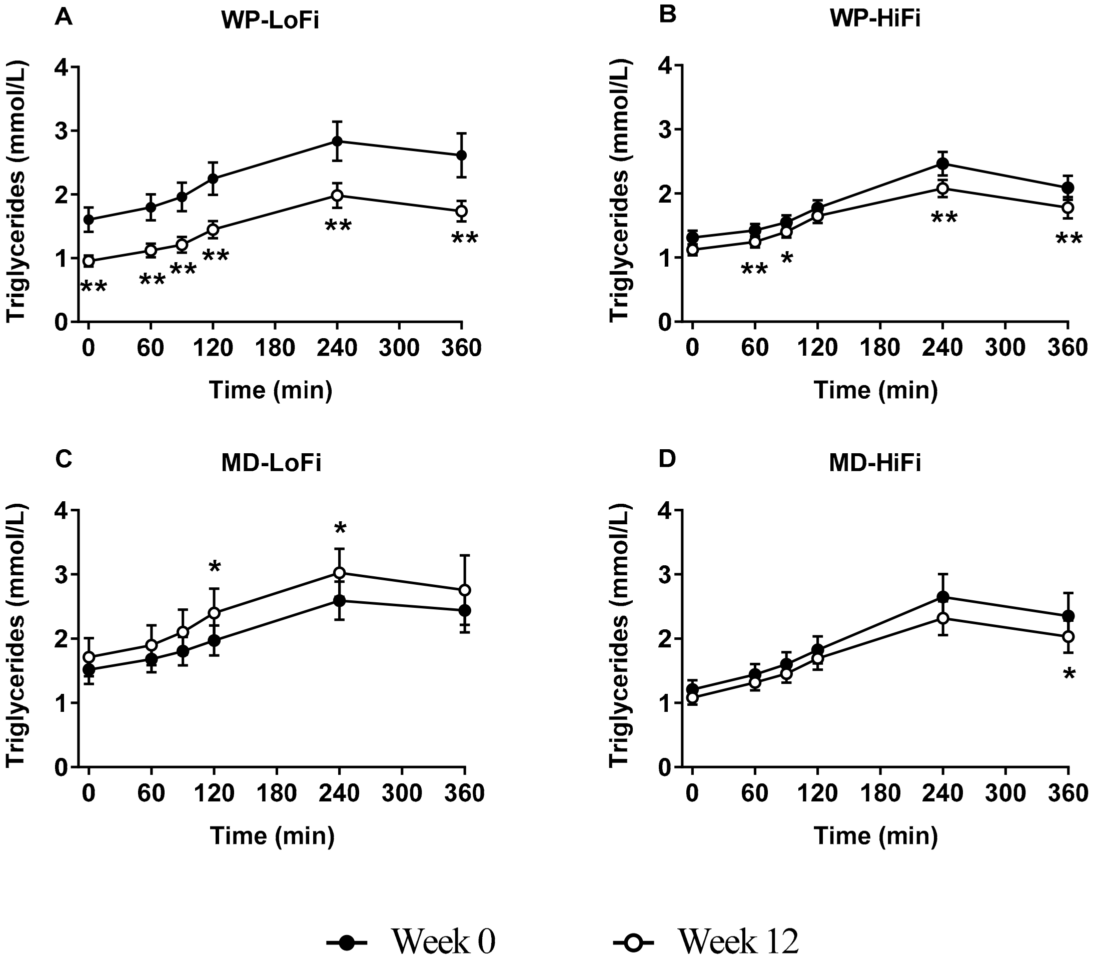

Values are means (SEM) at given time points. Significant difference between week 0 and week 12 at any given time point is indicated by * $P < 0.05$, ** $P < 0.01$. Analyzed by repeated-measures ANOVA. HiFi, high fiber; LoFi, low fiber; MD, maltodextrin; WP, whey protein.
